# Health problems and health care seeking behavior among adult backpackers while traveling in Thailand

**DOI:** 10.1186/s40794-016-0026-9

**Published:** 2016-06-22

**Authors:** Chayasin Mansanguan, Wasin Matsee, Peyawadee Petchprapakorn, Nujareenart Kuhakasemsin, Niracha Chinnarat, Jutarmas Olanwijitwong, Watcharapong Piyaphanee

**Affiliations:** 1 Department of Clinical Tropical Medicine, Travel Medicine Research Unit, Faculty of Tropical Medicine, Mahidol University, Bangkok, Thailand; 2 Hospital for Tropical Diseases, Faculty of Tropical Medicine, Mahidol University, Bangkok, Thailand

**Keywords:** Heath problems, Backpackers, Thailand

## Abstract

**Background:**

Health problems among travelers in developing countries are not uncommon. Little is known about the occurrence of health problems and their impacts among backpackers in Thailand. The objective of this study was to assess the health problems and the health seeking behavior among adult backpackers who visited Thailand.

**Methods:**

This was a cross-sectional questionnaire-based study. Data were collected from foreign adult backpackers in Khao San Road, Bangkok. They were asked about their demographic data, health problems (if any), and health-care seeking behavior if they became ill.

**Results:**

During May to July 2015, 420 backpackers were enrolled in this study. Of these, 51.4 % were male with a median age of 26.4 years. Up to 66.9 % were of European origin, while 17.4 % were North American. Fifty-eight percent sought pre-travel consultation before traveling. In this study, 10.2 % (43/420) of the participants reported at least health problem during travel. Most of them (79.1 %) had single episode of illness. Of this, diarrhea was the most common health problem (30.4 %), followed by abdominal pain (14.3 %), skin problems (8.9 %), respiratory problems (8.9 %), accident-associated injury (7.1 %), and febrile illness (7.1 %). One third of backpackers with health problems spontaneously recovered without any treatment, while nearly a quarter treated themselves with standby medication, and one in five had to buy over the counter medication. Just over 9 % of backpackers with a health problem sought medical care at a hospital. Among travellers with health problems, 72.1 % reported that their health problems did not significantly impact to their trip, while 23.3 % had to delay or postpone their trip for at least 1 day, and 4.7 % had to cancel some trip and/or activity. Longer duration of stay was significantly related to higher occurrence of health problems while traveling.

**Conclusions:**

Approximately 10 % of adult backpackers experienced some health problems during their trip in Thailand. Although most of their health problems were mild, up to 22 % of backpackers with health problems need to seek medical care either in a clinic or in a hospital.

## Background

Thailand is one of the main tourist destinations in Southeast Asia and the number of tourists continues to grow. According to the UNWTO (United Nation – World Tourism Organization), 19 million foreigners visited Thailand in 2010 and the number increased to 25 million in 2014 [1]. Unfortunately, in the same year (2014), there were 41,061 foreigners who were reported ill according to the reports from the Bureau of Epidemiology, Ministry of Public Health of Thailand [2]. Based on these numbers, the prevalence of notifiable health problems was 2.1 per 1000 travelers. However, it is clear that the true incidence of health problems is likely much higher since these numbers were based on the surveillance report from health care facilities throughout Thailand and likely under captures health care events. Unfortunately, this surveillance system is not designed to capture mild or self-limiting health problems.

Although several studies have reported the incidence of health problems among travelers in Thailand, they focused only on specific health problems such as diarrhea and animal bites [3–5]. These studies did not describe the true magnitude and impact of the variety of likely health problems encountered among travelers to Thailand. Therefore, in this study, we aimed to determine the prevalence and spectrum of health problems among backpackers in Thailand. We also aimed to assess their practices and the impact of health problem during their trips.

## Methods

This was a cross-sectional, questionnaire-based study. Data were collected from foreign backpackers in the Khao San Road area, a famous backpacker center in Bangkok, Thailand. Eligible participants were adult backpackers (aged ≥ 18 years), who could understand and were willing to complete the English language questionnaire. Foreign expatriates living or working in Thailand were excluded. A convenience sampling method was used in data collection. The investigator team invited any backpackers who was readily available in the study area. Verbal consent was obtained. Eligible participants were invited to complete the anonymous study questionnaire which was comprised of three parts including the demographic information and health problems during travel, practices of seeking medical care, and the impact of health problems to their trips.

Sample size was calculated by using the estimated number of backpackers in the Khao San Road area during the data collection period [6] and the Taro Yamane Standard sample size table [7]. In order to achieve 95 % confidential interval, at least 381 participants were required. Therefore, we planned to collect 400 questionnaires in this study.

### Statistical analysis

All statistical analyses were performed using SPSS for Windows (IBM Corp, Armonk, NY, USA). Continuous data were presented as mean with SD (for normally distributed data) or median with range (for non-normal distributed data). Categorical data were presented as numbers and percentage. The Student’s *t*-test was used to compare means of two groups, while the chi-square and Fisher’s exact test were used for categorical data, as appropriate. A *p-value* of < 0.05 was regarded as statistically significant.

## Results

Data were collected in the Khao San Road area during May to July 2015. Four hundred and twenty questionnaires were completed and analyzed. Fifty-one percent of travelers were male and the overall median age was 26.4 (range 18–68) years. Up to 66.9 % were of European country origin, while 17.4 % were form North American. The main reason for travel was tourism (95.5 %). Respondents reported that they planned to travel in Thailand for an average of 26.2 (range 1–365) days. Nearly 60 % (58.3 %) of travelers had sought pre-travel consultation before this trip in Thailand. The average travel duration of stay from arrival in Thailand to data collection was 10.2 (range 1–150) days. Detail demographic data is shown in Table 1.

**Table 1:** Baseline characteristics of the study population

	*n*	%
Median age (years) (range)	26.4 (18–68 years)	
Gender		
Male	216	51.4
Female	204	48.6
Nationality		
European	281	66.9
North American	73	17.4
Asian	44	10.5
Australian & New Zealander	12	2.9
Latin American	8	1.9
African	2	0.5
Travel purpose		
Tourism	401	95.5
VFR (Visiting friends and relatives)	5	1.2
Missionary/volunteer	6	1.4
Other	7	1.7
Pre-travel counseling before this trip		
Yes	245	58.3
No	175	41.7
Health problem during the trip		
No	377	89.8
Single episode	34	8.1
2 episodes	5	1.2
3 episodes	5	0.5
More than 3 episodes	2	0.5

### Health problems among backpackers in Thailand

About 10 % (43/420) of the study participants reported some sort of health problems during their stay (Table 2). Most of them (79.1 %) had only single episode of illness, while 11.6 % had two episodes of illnesses. Among 56 episodes of illness, diarrhea was the most common health problem (30.4 %), followed by abdominal pain without diarrhea (14.3 %), upper respiratory tract symptoms 8.9 %, and skin problem 8.9 %. One backpacker with dengue infection was found in our study.

**Table 2:** Spectrum of health problems among participants

Health problems (*n* = 56)	*n*	%
Diarrhea	17	30.4 %
Abdominal pain	8	14.3 %
URI (Upper respiratory tract infection)	5	8.9 %
Skin problem and wound	5	8.9 %
Fever (Dengue, *n* = 1)	4	7.1 %
Accident	4	7.1 %
Unspecified	3	5.4 %
Mosquito bite	3	5.4 %
Ear pain	2	3.6 %
Cystitis	2	3.6 %
Hangover	2	3.6 %
Muscle strain	1	1.8 %

### Management of health problems and its impact

Approximately one third of health problems were mild and recovered spontaneously. Twenty-four percent of participants with health problems needed to take medication they brought in their medical kit. Twenty percent of participants needed to buy over the counter medication while five participants needed a visit to a hospital. One participant with dengue infection needed hospitalization. However, overall impact of illness on their itinerary was low (Table 3). Up to 70 % of participants with health problems reported no significant impact to their trip, 23.3 % needed to postpone or delay their trips, while 4.7 % of participants needed to cancel some trips/activities.

**Table 3:** Management of health problems and its impacts

	*n*	%
Management of health problem (*n* = 54)		
Spontaneous recovery without medication	18	33.3
Took their own medication	13	24.1
Bought over-the-counter medication	11	20.4
Visited a doctor in a clinic	6	11.1
Visited a doctor in a hospital	5	9.3
Admitted to a hospital	1	1.9
Impacts of health problems to the trip (*n* = 43)		
No significant impact	31	72.1
Had to postpone some trips/activities	10	23.3
Had to cancel some trips/activities	2	4.7

### Comparison of participants with health problem group and healthy group

Bivariate analysis was done to determine factors associated with health problems. The mean duration of stay of participants with health problems were significantly longer than participants without health problem (23.1 vs 8.8 days, *p* < 0.001). There was no statistical difference between the two groups in all other factors including age, sex, nationality, purpose of travel and pre-travel health advice. Detailed analysis and cumulative attack rate are shown in Tables 4 and 5, respectively.

**Table 4:** Comparison between participants with health problems and healthy group

	With health problems (*n* = 43)	Healthy (*n* = 377)	*P value*
Mean age (years)	26.4	26.3	0.37
Duration of stay (days)	23.1	8.8	<0.001*
Gender			
Male	21 (9.7 %)	195 (90.3 %)	0.72
Female	22 (10.8 %)	182 (89.2 %)	
Nationality			
Asian	2 (4.5 %)	42 (95.5 %)	0.29
Non-Asian	41 (10.9 %)	335 (89.1 %)	
Travel purpose			
Tourism	42 (10.4 %)	360 (89.6 %)	1.00
Non-tourism	1 (5.6 %)	17 (94.4 %)	
Pre-travel counselling			
Yes	29 (11.8 %)	216 (88.2 %)	0.20
No	14 (8.0 %)	161 (92.0 %)	

*Statistical significance

**Table 5:** Cumulative attack rate of any health problems and GI(Gastrointestinal) problems

Duration of stay in SEA	*n*	Cumulative attack rate	
		Any health problems	GI problems
Up to 7 days	279	3.94 %	2.51 %
Up to 14 days	325	6.46 %	3.08 %
Up to 21 days	360	7.50 %	3.89 %
Up to 28 days	374	8.29 %	4.55 %
All duration of stay (1–150 days)	420	10.24 %	5.00 %

## Discussion

In our study, 10.2 % of backpackers developed some sort of health problem in Thailand during an average stay of 10 days. This prevalence seems to be low when compared to other previous studies that showed the self-reported illness rate between 25–70 % among travelers in developing countries [5, 8–10]. It was not possible to compare our illness rate directly with other previous studies since there were major differences in terms of population studied, trip characteristic, destination, and duration of stay. However, several important points were noted. Firstly, most of the previous studies collected the data when travelers had already finished their trip not during the trips as was described in this study. So the average duration of stay in those studies were much longer. In a study among Israeli travelers, reported the illness rate up to 70 % in the average stay of 3.5 months [9]. A study in Germany reported a 42.9 % illness rate among returning travelers with an average trip duration of 23.9 days [10]. While in our study, we reported a health problem rate of 10.2 % during 10 days stay. Had we survey travelers at the end of their trip (average planned duration of 26 days), the health problem rate would have been considerably higher.

In our study, travelers’ diarrhea was the most common health problem found in participants. It was similar to other previous studies conducted among travelers returning from Southeast Asia [5, 10, 11]. However, the attack rate of diarrhea among travelers in Thailand seems to be lower over time. In 2009, we reported a 30.7 % attack rate of diarrhea among backpackers in Thailand [4]. While in 2011, we conducted an airport study (*n* = 7894), the attack rate of diarrhea was 16.1 % in the average stay of 28 days [12]. In the current study the attack rate of diarrhea was only 4 % during an average stay of 10 days. Although this is not conclusive evidence, it might suggest a decreasing trend. Our observation is not unique; several studies including a large cohort study have also reported a substantial lower attack rate of travelers’ diarrhea in many parts of the world [13].

As found in several studies, most illness episodes among travelers were mild with spontaneous recovery [5, 9, 14]. Only 2.14 % (9/420) of our participants required a doctor visit either in a clinic or a hospital during their trip, and one case needed to be hospitalized due to dengue infection. Although the attack rate of dengue in our study was not high, it cannot be neglected in pre-travel counseling for travelers to Southeast Asia, where it is still an important threat and fatal cases among travelers are occasionally reported [15, 16]. In this study, pre-travel counseling rate among backpackers was 59 %. It was relatively similar to previous study in Khao San Road area in 2012 [17].

This study had several limitations. First, we collected data among backpackers while travelling and their average duration of stay was only 10 days in Thailand and thus it was no possible to capture the whole spectrum of diseases, especially those with longer incubation period such as malaria or hepatitis. Because of our sampling method we were not able to estimate per-trip attack rates. However, we may potentially, represent the early health problems that occur in the first 10 days, and this could be considered as a strength. Since we collected after they have finished their trips, it would be much more difficult for them to recall some health problems they developed especially minor problems such as hangover that was reported in our study.

Second, the data was collected exclusively among backpackers in the Khao San Road area. Data from any single site could not represent the total population of backpackers who might stay in different areas and might have different travelling patterns or exposures. However, Khao San Road is a hub and it is the most famous backpacker center in Thailand. Thus, results from this area could give us some insight about risk and health seeking behavior practices.

## Conclusions

Approximately 10 % of backpackers experienced some health problems during their first 10 days in Thailand. Although most of their health problems were mild and recovered spontaneously, up to 22 % of backpackers with health problems needed to seek medical care in either a clinic or a hospital.

## Abbreviations

UNWTO, United Nation – World Tourism Organization; URI, upper respiratory tract infection; VFR, visiting friends and relatives

## References

[1] World Tourism Organization. UNWTO Tourism Highlights,2015 Edition. UNWTO. 2014. Available from: http://www.e-unwto.org/doi/pdf/10.18111/9789284416899. Accessed 10 Apr 2016.

[2] Bureau of Epidemiology, Department of Disease Control. Report of diseases surveillance in foreigners 2014. Article in Thai. Available from http://www.boe.moph.go.th/Annual/AESR2014/aesr2557/Part%201/1-8/foreigners.pdf Accessed 10 Apr 2016.

[3] Piyaphanee W, Shantavasinkul P, Phumratanaprapin W, Udomchaisakul P, Wichianprasat P, Benjavongkul M, Ponam T, Tantawichian T (2010). Rabies exposure risk among foreign backpackers in Southeast Asia. Am J Trop Med Hyg.

[4] Piyaphanee W, Kusolsuk T, Kittitrakul C, Sutthithum W, Ponam T, Wilairatana P (2011). Incidence and impact of travelers’ diarrhea among foreign backpackers in Southeast Asia: A result from Khao San Road, Bangkok. J Travel Med.

[5] Piyaphanee W, Kittitrakul C, Lawpoolsri S, Tangkanakul W, Sa-Ngiamsak N, Nasok P (2014). Incidence and spectrum of health problems among travelers to Laos. J Travel Med.

[6] Matichon online news. Increase tourism expected in Khao San Road. [Article in Thai]. Available from: http://www.matichon.co.th/news_detail.php?newsid=1422512311.

[7] Yamane T (1973). Sample size table in statistics: an introductory analysis.

[8] Hill DR (2000). Health problems in a large cohort of Americans traveling to developing countries. J Travel Med.

[9] Winer L, Alkan M (2002). Incidence and precipitaing factors of morbidity among Israeli travelers abroad. J Travel Med.

[10] Rack J, Wichmann O, Kamara B, Gunther M, Cramer J, Schonfeld C, Henning T, Schwarz U, Muhlen M, Weitzel T, Friedrich-Janicke B, Foroutan B, Jelinek T (2005). Risk and spectrum of diseases in travelers to popular tourist destinations. J Travel Med.

[11] Kuda K, Mizuno Y (2009). Travel-related health problems in Japanese travelers. Travel Med Infect Dis.

[12] Kittitrakul C, Lawpoolsri S, Kusolsuk T, Olanwichitwong J, Tangkanakul W, Piyaphanee W (2015). Travelers’ Diarrhea in Foreign Travelers in Southeast Asia: A Cross-Sectional Study in Bangkok, Thailand. Am J Trop Med Hyg.

[13] Pizurra R, Steffen R, Tschopp A, Mutsch M (2010). Diarrhea in a large prospective cohort of European travellers to resource-limited destinations. BMC Infect Dis.

[14] Abdullah ASM, Hamer DH (2006). Travel-related health problems of Hong Kong residents: Assessing the need for travel medicine services. Travel Med Infect Dis.

[15] Waagsbø B, Sundøy A, Høyvoll LR (2010). Febrile illness in a returned traveller from Thailand. J Clini Virol.

[16] Huhtamo E, Vuorinen S, Uzcátegui NY, Vapalahti O, Haapasalo H, Lumio J (2006). Fatal dengue virus infection in a Finnish traveler. J Clin Virol.

[17] Kaehler N, Piyaphanee W, Kittitrakul C, Kyi YP, Adhkari B, Sibunruang S, Jearraksuwan S, Tangpukdee N, Silachamroon U, Tantawichian T (2013). Sexual behavior of foreign backpackers in the Khao San Road area, Bangkok. Southeast Asian J Trop Med Public Health.

